# Appraisals of psychotic experiences: an experimental investigation of symptomatic, remitted and non-need-for-care individuals

**DOI:** 10.1017/S0033291715002780

**Published:** 2016-01-25

**Authors:** R. Underwood, V. Kumari, E. Peters

**Affiliations:** 1 Department of Psychology, King's College London, Institute of Psychiatry, Psychology and Neuroscience, London, UK; 2 National Institute for Health Research (NIHR) Biomedical Research Centre for Mental Health at South London and Maudsley NHS Foundation Trust, London, UK

**Keywords:** Anomalous experiences, appraisal, need-for-care, psychosis

## Abstract

**Background:**

Appraisals are suggested to play a determining role in the clinical outcome of psychotic experiences (PEs). We used experimental tasks that mimic PEs to investigate appraisals in individuals with PEs with and without a ‘need-for-clinical-care’, and psychosis patients whose symptoms have remitted. We predicted that patients would appraise the tasks as threatening regardless of current symptom level, while non-clinical and control groups would appraise them as non-threatening.

**Method:**

Appraisals following three anomalous experiences-inducing tasks [Telepath, Cards task, Virtual acoustic space paradigm (VASP)] were examined in 71 individuals: symptomatic (*n* = 18) and remitted (*n* = 16) psychosis patients; non-clinical group with PEs (*n* = 16); controls without PEs (*n* = 21).

**Results:**

As predicted, symptomatic patients endorsed more threatening appraisals for all tasks than non-clinical and control groups, who did not differ from each other. However, remitted patients were less likely to endorse threatening appraisals of the Cards and Telepath than their symptomatic counterparts, although they did not differ in global ratings of how striking, threatening and distressing they found the tasks. Moreover, remitted participants endorsed more threatening appraisals of the Telepath and VASP than non-clinical participants, and of the VASP than controls. Remitted participants also rated all three tasks as globally more threatening than the non-clinical group and controls.

**Conclusions:**

Clinical outcome may not necessarily be driven by the presence of symptoms, with threatening appraisals of PEs representing a key factor. The remitted group's intermediate appraisal scores imply that the relationship between appraisal and clinical outcome is not straightforward, and potential mediating factors need to be determined.

## Introduction

Recent reviews and meta-analyses show a continuity of psychosis in the general population that includes healthy states (Van Os *et al.* 2009; Linscott & Van Os, 2013). Cognitive models of psychosis suggest that appraisals can be distinguished or ‘decoupled’ from actual psychotic experiences (PEs), and play a key role in the transition to a clinical ‘need-for-care’ (Bentall *et al.* 2001; Garety *et al.* 2001, 2007; Morrison, 2001; Bentall *et al.* 2007; Howes & Murray, 2014). According to Garety *et al.* (2001), maladaptive appraisals are those in which PEs are attributed to an external cause and seen as personally significant. These appraisals in turn may lead to symptom-associated distress, resulting in the individual needing treatment.

A minority of individuals report persistent PEs but have never sought or been in need of treatment (Linscott & Van Os, 2013). These individuals form a valuable comparison group for clinical participants, as although their experiences tend to be less frequent, they are of equivalent phenomenology, but differ in outcome (Peters *et al.* in press). Using an in-depth interview [Appraisals of Anomalous Experiences Interview (AANEX); Brett *et al.* 2007], we previously found that non-need-for-care individuals normalize their experiences as psychologically explainable phenomena, or integrate them within a spiritual or paranormal framework (Brett *et al.* 2007; Lovatt *et al.* 2010), which in turn is predictive of less distress (Brett *et al.* 2014).

These studies also demonstrated that externally attributed experiences are not necessarily maladaptive, since non-need-for-care participants also attribute their PEs to external, albeit benevolent, causes such as spiritual guidance (Brett *et al.* 2007). Rather, group differences concerned attributions of danger, emotional valence and agency, with clinical participants typically viewing their experiences as caused by other people who wish them harm. Ultimately, the core of maladaptive appraisals contributing to a need-for-care relate to threat. Cognitively, non-need-for-care individuals may have decoupled threatening appraisals from their PEs.

Recently, these differences in appraisals between those with and without a need-for-care have been replicated using anomalous experience-inducing tasks (Linney & Peters, 2007; Taylor *et al.* 2013; Ward *et al.* 2014). Employing tasks that mimic PEs provides each participant with an identical experience, enabling variation in appraisals to be disentangled from variation in the phenomenology and content of PEs.

To date, studies have compared non-clinical with clinical participants with ongoing PEs, but not with clinical participants whose symptoms have remitted. Cognitive models of psychosis would predict that clinical individuals would retain a threat-based appraisal style even when their symptoms have remitted, since appraisal style can be disentangled from PEs themselves, with a range of potential risk factors contributing to threatening appraisals independently of the presence of PEs (Garety & Freeman, 2013). Indeed, recent evidence showed that patients displayed a personalizing bias (i.e. blaming other people for a negative event) regardless of whether their paranoia symptoms were acute or remitted (Berry *et al.* 2014).

The present study sought to replicate and extend previous findings, first by comparing appraisals of experimentally induced anomalous experiences across four groups: symptomatic and remitted psychosis patients, those with PEs but without a need-for-care, and controls without PEs. Second, a new anomalous experience-inducing task, the ‘Telepath’, was administered in addition to the Cards and Virtual acoustic space paradigm (VASP) tasks (previously used in Ward *et al.* 2014). It was predicted that patients, regardless of symptom level, would appraise the tasks as more threatening than those without a need-for-care and controls, who would not differ from each other. It was also predicted that symptomatic and remitted patients would exhibit equivalent appraisal scores.

## Method

The study was approved by Dulwich Research Ethics Committee (13/LO/0390).

### Participants

#### Clinical groups

The 18 symptomatic and 16 remitted participants were patients with a psychosis spectrum disorder [International Classification of Diseases (ICD)-10 diagnoses F20–F39]. To distinguish symptomatic and remitted groups, symptomatic patients all had a current score ⩾3 (hallucination occurring at least weekly/delusional belief with high conviction) on one or more items of the Scale for the Assessment of Positive Symptoms (SAPS; Andreasen, 1984), while remitted patients all had previously experienced positive psychotic symptoms, but in the last month scored ⩽2 (hallucination occurring at most monthly/simple delusions that are questioned) on any SAPS item. Patients were recruited from in-patient wards,out-patient recovery services, service-user led organizations and a psychological therapies service (Psychological Interventions Clinic for out-patients with Psychosis; PICuP) research register, all in the South London and Maudsley NHS Foundation Trust.

#### Non-clinical group

A total of 16 individuals displaying PEs but without a need-for-care (i.e. had never received a diagnosis, or been in need of mental health services for their experiences) were recruited. Individuals were screened for PEs on the Unusual Experiences Screening tool (see below), and only those with a score ⩾3 on one or more SAPS items (to match the symptomatic group), in the absence of drug use and in clear consciousness, and whose experiences started more than 5 years previously (to avoid prodromal individuals), participated. Anyone scoring 2 (‘unmet need’) on the ‘psychological distress’ (in relation to PEs) and ‘self-care’ dimensions of the Camberwell Assessment of Need Short Appraisal Schedule (CANSAS; Slade *et al.* 1999) were excluded. Non-clinical participants were recruited from specialist sources using a sampling strategy developed in previous studies (Heriot-Maitland *et al.* 2012; Ward *et al.* 2014). None had ever had contact with secondary care mental health services, but two had received treatment for depression from their general practitioner in the past.

#### Control group

A total of 21 controls scoring within 1 standard deviation of the population mean (15) or lower on the unusual experiences subscale of the Oxford–Liverpool Inventory of Feelings and Experiences (O-LIFE; Mason *et al.* 1995) were recruited. Online advertisements were distributed via a circular email list internal to King's College London, local online forums and the ‘Experimatch’ online research register. None had ever had contact with primary or secondary care services for mental health problems.

#### All groups

All participants were required to have normal or corrected vision and hearing.

#### Exclusion criteria

Exclusion criteria were: insufficient command of English; neurological history, head injury or epilepsy; primary substance dependence; estimated intelligence quotient (IQ) < 70 [estimated from Wechsler Test of Adult Reading (WTAR); Wechsler, 2001]. A history of common mental disorders was not an exclusion criterion for any group. Participants were excluded if they had previously seen the anomalous experience-inducing tasks.

The sample demographic and clinical characteristics are presented in Tables 1 and 2, including group differences (all of which were in line with previous findings in these groups; Peters *et al.* in press).

**Table 1.** Sample characteristics and statistical differences between the symptomatic, remitted, non-clinical and control groups

	Characteristics	C-S (*n* = 18)	C-R (*n* = 16)	NC (*n* = 16)	Controls (*n* = 21)	Significance tests
Gender, *n*	Male Female	13 5	8 8	5 11	7 14	*χ*^2^_3_ = 7.826, *p* = 0.050 (*V* = 0.33) C-S: male > female
Mean age, years (s.d.)		42.56 (12.03)	43.75 (11.95)	52.5 (9.25)	30.05 (10.32)	*F*_3,67_ = 13.35, *p* < 0.001 (*η*_p_^2^ = 0.37)^f^^***,^ ^i^^**^
Ethnicity, *n*	Whites	7	11	14	15	*χ*^2^_6_ = 12.76, *p* = 0.047 (*V* = 0.30)
	Non-whites	11	5	2	6	C-S: non-white > white
Employment, *n*	Employed/in education	4	7	15	17	*χ*^2^_3_ = 23.11, *p* < 0.001 (*V* = 0.57) NC and controls: employed > unemployed;
	Not employed	14	9	1	4	C-S: unemployed > employed
Mean time in education, years (s.d.)^a^		15.12 (3.76)	15.07 (3.73)	22.5 (8.00)	18.95 (7.05)	*F*_3,64_ = 5.46, *p* = 0.002 (*η*_p_^2^ = 0.20)^d^^**,^ ^e^^**^
Highest level of education, *n*	University education	7	5	11	15	*χ*^2^_3_ = 17.31, *p* = 0.001 (*V* = 0.49) NC: university > no university
	No university education	11	11	5	6	
Mean WTAR (s.d.)	Predicted full-scale IQ	102.33 (10.19)	103.31 (8.25)	109.31 (3.50)	111 (4.74)	*F*_3,67_ = 6.69, *p* = 0.001 (*η*_p_^2^ = 0.23)^d^^*,^ ^g^^**,^ ^h^^*^
Religious affiliation, *n*	Traditional	15	7	7	5	*χ*^2^_6_ = 18.25, *p* = 0.004, (*V* = 0.36)
	Other/spiritual	1	2	2	1	C-S: traditional > no affiliation
	None	2	7	7	16	
Mean DASS-21 (s.d.)	Depression	6.56 (4.78)	7.25 (6.56)	0.69 (1.08)	1.57 (1.63)	*F*_3,67_ = 11.84, *p* < 0.001 (*η*_p_^2^ = 0.35)^i^^**,^ ^j^^***,^ ^k^^***,^ ^l^^***^
	Anxiety	5.72 (5.30)	6.75 (5.94)	0.94 (1.18)	0.90 (1.37)	*F*_3,67_ = 10.57, *p* < 0.001 (*η*_p_^2^ = 0.32)^i^^**,^ ^j^^**,^ ^k^^***,^ ^l^^**^
	Stress	7.67 (6.16)	9.31 (6.03)	2.69 (2.09)	3.14 (3.51)	*F*_3,67_ = 8.32, *p* < 0.001 (*η*_p_^2^ = 0.27)^i^^*,^ ^j^^*,^ ^k^^**,^ ^l^^**^
Parental occupation, *n*	Professional/intermediate	13	10	12	16	*χ*^2^_3_ = 2.17, *p* = 0.54 (*V* = 0.18)
	Other	5	6	4	4	
	Missing value	0	0	0	1	
Children, *n*	Yes	3	6	11	2	*χ*^2^_3_ = 17.24, *p* = 0.001 (*V* = 0.49)
	No	15	10	5	19	NC: children > no children
Diagnosis by ICD-10		Schizophrenia = 14 (78%) Schizo-affective = 2 (11%) Psychosis NOS = 0 F30–F39 = 2 (11%)	Schizophrenia = 6 (37.5%) Schizo-affective = 1 (6.25%) Psychosis NOS = 1 (6.25%) F30–F39 = 8 (50%)	–	–	Schizophrenia: C-S > C-R F30–F39: C-R > C-S

Antipsychotic medication and dosages		Medicated = 17 (94.4%) None = 1 (5.6%) Typical = 5.6% Atypical = 66.7% Clozapine = 16.7% More than 1 = 11.1% Median = 50% maximum daily recommended dose (range = 17–100%)^b^	Medicated = 10 (62.5%) None = 6 (37.5%) Typical = 0% Atypical = 37.5% Clozapine = 12.5% More than 1 = 0% Median = 39% maximum daily recommended dose (range = 5– 100%)^c^	–	–	*χ*^2^_1_ = 3.70, *p* = 0.054 (*V* = 0.33) On medication: C-S > C-R
	
Mean number of admissions [median] (range)		5.59 [4] (0–17)	2.69 [2] (0–10)	–	–	*U* = 51.5, *p* = 0.012 (*d* = 0.64)
Mean time since onset, years (s.d.)		20.47 (13.15)	19.20 (14.16)	38.07 (15.44)	–	*F*_2,43_ = 8.02, *p* = 0.001 (*η*_p_^2^ = 0.27)^d^^**,^ ^e^^**^

C-S, Symptomatic, C-R, remitted, NC, non-clinical; s.d., standard deviation; WTAR, Wechsler Test of Adult Reading; IQ, intelligence quotient; DASS-21, 21-item Depression Anxiety and Stress Scales; ICD, International Classification of Diseases; NOS, not otherwise specified.

a One value was missing for C-S, and two for C-R.

b Six participants had missing data for dosage.

c One participant had missing data for dosage.

d NC *v*. C-S.

e NC *v*. C-R.

f NC *v*. controls.

g Controls *v*. C-S.

h Controls *v*. C-R.

i C-S *v*. controls.

j C-S *v*. NC.

k C-R *v*. controls.

l C-R *v*. NC.

* *p* < 0.05, ** *p* < 0.01, *** *p* < 0.001 (Tukey's least significant difference test).

**Table 2.** Summary of clinical measure scores by group and statistical differences between symptomatic, remitted and non-clinical groups^a^

				C-S *v*. NC
	C-S (*n* = 18)	C-R (*n* = 16)	NC (*n* = 16)	NC *v*. CR
SAPS hallucinations	3.72 (1.71)	0.44 (0.81)	2.75 (1.53)	*U* = 206.5, *p* = 0.030 (*d* = 0.43)
				*U* = 33, *p* < 0.001 (*d* = 0.74)
SAPS delusions	3.67 (1.24)	0.69 (0.95)	2.81 (0.75)	*U* = 227.5, *p* = 0.003 (*d* = 0.58)
				*U* = 11, *p* < 0.001 (*d* = 0.91)
SAPS thought disorder	1.0 (1.41)	0	0.19 (0.54)	*U* = 186.5, *p* = 0.144 (*d* = 0.30)
				*U* = 112, *p* = 0.564 (*d* = 0.12)
SAPS bizarre behaviour	0.78 (1.11)	0.13 (0.50)	0.06 (0.25)	*U* = 193.5, *p* = 0.088 (*d* = 0.34)
				*U* = 128.5, *p* = 1.00 (*d* = 0.00)
SAPS inappropriate affect	0.17 (0.71)	0	0	*U* = 152, *p* = 0.798 (*d* = 0.05)
				*U* = 128, *p* = 1.00 (*d* = 0.00)
SANS affective flattening	1.17 (1.25)	0.31 (0.87)	0	*U* = 224, *p* = 0.005 (*d* = 0.55)
				*U* = 144, *p* = 0.564 (*d* = 0.13)
SANS alogia	1.28 (1.27)	0.06 (0.25)	0	*U* = 224, *p* = 0.005 (*d* = 0.55)
				*U* = 136, *p* = 0.780 (*d* = 0.06)
SANS avolition	2.56 (1.69)	1.44 (1.71)	0	*U* = 256, *p* < 0.001 (*d* = 0.78)
				*U* = 192, *p* = 0.015 (*d* = 0.50)b
SANS anhedonia	0.76 (2.05)	1.00 (1.51)	0	*U* = 216, *p* = 0.012 (*d* = 0.50)
				*U* = 176, *p* = 0.073 (*d* = 0.38)
SANS attention	2.61 (1.69)	2.61 (1.69)	1.63 (1.46)	*U* = 194.5, *p* = 0.081 (*d* = 0.35)
				*U* = 145, *p* = 0.539 (*d* = 0.13)
AANEX total – lifetime experiences	33.94 (9.31)	32.75 (8.78)	38.13 (3.95)	*U* = 115.5, *p* = 0.330 (*d* = 0.20)
				*U* = 89, *p* = 0.149 (*d* = 0.30)
AANEX total – current experiences	31.28 (7.93)	21.44 (3.97)	35.06 (4.36)	*U* = 95.5, *p* = 0.095 (*d* = 0.34)
				*U* = 3.5, *p* < 0.001 (*d* = 0.97)
Total AANEX score	63.22 (16.00)	52.25 (10.68)	70.19 (7.11)	*U* = 109.5, *p* = 0.237 (*d* = 0.24)
				*U* = 19, *p* < 0.001 (*d* = 0.85)
AANEX – meaning–reference – current	6.83 (2.23)	4.88 (1.26)	10.44 (1.97)	*U* = 35.5, *p* < 0.001 (*d* = 0.75)
				*U* = 5, *p* < 0.001 (*d* = 0.96)
AANEX – ‘paranormal–hallucinatory’ – current	4.94 (1.63)	3.19 (0.54)	6.50 (1.41)	*U* = 74.5, *p* = 0.015 (*d* = 0.48)
				*U* = 3, *p* < 0.001 (*d* = 0.98)
AANEX – ‘cognitive–attention’ – current	4.78 (2.16)	3.75 (1.61)	3.19 (0.54)	*U* = 205, *p* = 0.036 (*d* = 0.42)
				*U* = 139, *p* = 0.696 (*d* = 0.09)
AANEX – ‘dissociative–perceptual’ – current	4.39 (1.58)	3.31 (0.70)	4.38 (1.78)	*U* = 148, *p* = 0.905 (*d* = 0.03)
				*U* = 82.5, *p* = 0.86 (*d* = 0.36)
AANEX – first rank symptoms – current	10.44 (2.55)	5.88 (1.41)	10.06 (1.53)	*U* = 157.5, *p* = 0.646 (*d* = 0.09)
				*U* = 7.5, *p* < 0.001 (*d* = 0.94)

Data are given as mean (standard deviation).

C-S, Symptomatic; C-R, remitted; NC, non-clinical; SAPS, Scale for the Assessment of Positive Symptoms (Andreasen, 1984); SANS, Scale for the Assessment of Negative Symptoms (Andreasen, 1983); AANEX, Appraisals of Anomalous Experiences Interview (Brett *et al.* 2007).

a All scores for SAPS and SANS items are global scores.

b C-R significantly higher than NC.

### Measures

#### SAPS (Andreasen, 1984) and Scale for the Assessment of Negative Symptoms (SANS; Andreasen, 1983)

SAPS and SANS are interviews assessing positive/negative symptoms over the last month, rated on a six-point scale (0–5), and have shown good reliability and internal consistency (Andreasen & Grove, 1986; Mance & Haas, 1994).

#### Unusual Experiences Screening Questionnaire [UESQ; derived from the Appraisals of Anomalous Experiences Interview (Brett *et al.* 2007) and the Psychosis Screening Questionnaire (Bebbington & Nayani, 1995)]

The two screening measures were merged to avoid repetition of items. The UESQ assesses the presence of positive and first-rank psychotic symptoms within the last month and in the absence of drug use and in clear consciousness.

#### CANSAS (Slade *et al.* 1999)

The CANSAS was used to determine any unmet needs relating to underlying mental illness in the non-clinical group. Items 1–4 (covering basic self-care) and 9 (‘psychological distress’: ‘have you recently felt very sad or low in relation to PEs?’) were used. Scores for each item are: 0 = no problem; 1 = met need; 2 = unmet need; and 9 = not known.

#### O-LIFE (Mason *et al.* 1995)

The O-LIFE measures psychosis-proneness. The 30-item unusual experiences subscale (assessing mild forms of anomalous experiences such as having vivid daydreams) was used to screen the control group. The O-LIFE has shown good validity and reliability (Mason & Claridge, 2006).

#### AANEX – short form (Lovatt *et al.* 2010)

The short-form AANEX-Inventory consists of 17 items covering five factors: ‘meaning–reference’ (e.g. ideas of reference); ‘paranormal–hallucinatory’ (e.g. visual or somatic hallucinations); ‘cognitive–attention’ (e.g. thought block); ‘dissociative–perceptual’ (e.g. depersonalization); and ‘first-rank symptoms’ (e.g. hearing voices). There are three items per factor, except ‘meaning–reference’ and ‘first-rank symptoms’ factors which have four items each. Each item is rated for the lifetime presence of the experience, and within the last month, as ‘not present’ (1), ‘unclear’ (2) or ‘present’ (3). Factor scores are obtained by summing individual item scores (range of scores for each factor: 3–9, except ‘meaning–reference’ and ‘first-rank symptoms’ where the range is 4–12). Total scores range from 17 to 51. The AANEX has demonstrated good reliability and construct validity (Brett *et al.* 2007).

#### 21-Item Depression Anxiety and Stress Scales (DASS-21; Lovibond & Lovibond, 1995)

The DASS-21 is a 21-item self-report questionnaire assessing depression, anxiety and stress over the previous week, with seven items in each subscale. Each item is scored from 0 (did not apply to me at all) to 3 (applied to me very much or most of the time). Scores for each subscale range between 0 and 21, and total score between 0 and 61. The DASS-21 has demonstrated good validity for measuring the dimensions of depression, anxiety and stress (Henry & Crawford, 2005).

#### The WTAR (Wechsler, 2001)

The WTAR is a measure of pre-morbid IQ consisting of 50 irregularly spelled words that the participant is asked to pronounce sequentially. There is evidence that the WTAR is robust in the context of low effort (Whitney *et al.* 2010).

### Anomalous experiences analogue tasks^1^†

#### Cards task (http://sprott.physics.wisc.edu/pickover/esp2.html; Linney & Peters, 2007)

This task was used as an ‘analogue’ of thought interference symptoms. This card trick gives the impression that a computer has been able to read the participant's mind. Participants are shown six playing cards (face cards only) on a computer, and are asked to memorize one. They are then informed that their card will be selected and removed. They are subsequently shown five different cards for 3 s. This trick relies on the fact that people only scan for the card they have chosen and do not notice that all the cards have been replaced with similar but different face cards. This process was repeated five times.

#### Telepath phone application (http://richardwiseman.wordpress.com/video-audio/can-you-figure-out-the-secret-of-telepath/)

This ‘mindreading’ task was also an analogue of thought interference, using a smartphone application, presented via webcam on a computer screen. Four numbers (1–4) are presented to the participant who is required to mentally choose one number. Following the phone being placed face down (shaking the phone in the process), the participant is asked to reveal their choice to the experimenter. Unknown to the participant, shaking the phone activates an animation, cycling through each number consecutively, with each transition signalled by a sparkle sound every 8 s, enabling the experimenter to keep track. When the phone is lifted up by the experimenter the animation freezes and ‘magically’ reveals the chosen number. This process is repeated six times (see Appendix 1 for further description).

#### VASP (Wightman & Kistler, 1989)

This task was designed to be an ‘analogue’ of auditory hallucinations or ‘loud thoughts’ (Ward *et al.* 2014). The VASP allows sounds to be perceived as externally located through acoustic manipulation via computer software, despite presentation via headphones (for a detailed description of the acoustic manipulation process, see Ward *et al.* 2014). Participants are informed that the task assesses the effects of distraction on performance, as they are asked to complete a distractor task (determining the presence of objects in fuzzy images) while listening to the headphones. The participant's name is recorded beforehand, along with neutral commands (‘listen up’, ‘pay attention’, ‘concentrate’), heard as if originating from ‘outside the head’. These recordings are then played back at random intervals over a soundtrack of white noise (heard ‘inside the head’).

### Assessment of appraisals

Following each task, spontaneous explanations for the anomalous experiences were elicited to determine if the manipulation had been guessed correctly. Subsequently, participants completed a computerized rating scale (0–10) asking them to rate their conviction in a number of predetermined possible explanations. The explanations were taken from Ward *et al.* (2014), reflecting the most relevant appraisal styles ascertained in previous studies, namely normalizing, personalizing, intentionalizing, generalizing and externalizing/internalizing (Brett *et al.* 2007; Linney & Peters, 2007). The seven explanations used and their corresponding appraisal styles, categorized into ‘threatening’ and ‘non-threatening’, are summarized in Table 3. A further three visual analogue scales were used to assess globally how striking, distressing and threatening the participants found the tasks.

**Table 3.** Threatening and non-threatening appraisal styles in the three experimental tasks

	Cards task	Telepath task	VASP
Non-threatening appraisals			
External – normalizing	‘It is just a simple card puzzle’	‘It is just a simple number puzzle’	‘It is part of the study and involves a pre-recorded voice’
Internal – normalizing	‘It is to do with natural extrasensory perception (ESP)/psychic or paranormal abilities’		
	‘There is a rational explanation involving basic attention/perception’		
Threatening appraisals			
External – personalizing	‘It is not the computer which guessed; there is someone involved in this’	‘It was not just about the phone; there is someone behind the scenes involved’	‘Someone was speaking to me’
External – non-personalizing	‘It works because the system is able to read people's minds’		‘There was a spirit or some kind of entity in the room’
External – intentionalizing	‘It was done on purpose to trick me, or make me look stupid’		
External – generalizing	‘It is a trick that is part of a bigger conspiracy’		
Internal – non-normalizing	‘This means that something is wrong with me’		

VASP, Virtual acoustic space paradigm.

### Apparatus

All tasks were presented on a laptop. Speech for the VASP was recorded through the laptop soundcard using the software programme Cool Edit Pro (Syntrillium Software Corporation, 2003), recording at 16 000 Hz 16 bit mono. The computer audio output level was set at 28/50, with the white noise attenuated to −14 Db and the voice files to −10 Db within the VASP's Graphical User Interface (GUI). All tasks were programmed in Visual Basic.NET and were operated via a GUI.

### Procedure

Presentation of the three tasks was pseudo-randomized to control for order effects. The remaining measures were presented in such a way as to limit the effects of fatigue on performance, e.g. more challenging or lengthy measures were presented nearer the beginning of the session. Upon completion of the study, participants were debriefed and compensated for their time and travel.

### Data analysis

Data analysis was carried out using SPSS for Windows (version 22, 2013). The *α*-level of significance (two-tailed) was set at *p* < 0.05 unless indicated otherwise. Appraisals were split into ‘non-threatening’ and ‘threatening’ for analysis (see Table 3). Appraisal ratings from participants who guessed the true nature of the tasks were included, on the grounds that they could be considered as non-threatening appraisals, that happened to be correct. We previously carried out sensitivity analyses in a larger sample to examine whether group differences in appraisals of the same tasks were affected by the inclusion or exclusion of correct guesses (Peters *et al.* 2015), and found that observed group differences remained the same. Appraisal data were not normally distributed, and were analysed using non-parametric statistics. Main effect of group was analysed using a Kruskal*–*Wallis *H* test, followed by Mann–Whitney *U* tests for individual group comparisons. Effect size estimates were calculated using Cliff's delta (Cliff, 1993), a robust, non-parametric equivalent to Cohen's *d* (Hess & Kromrey, 2004). The thresholds of magnitude for Cliff's delta are: *d* < 0.147 ‘negligible’, *d* < 0.33 ‘small’, *d* < 0.474 ‘medium’, otherwise ‘large’ (Romano *et al.* 2006).

## Results

Threatening and non-threatening appraisals correlated highly across all three tasks (Table 4), showing good reliability for the tasks to elicit similar responses within each participant.

**Table 4.** Non-parametric correlations between appraisal scores (separated into threatening and non-threatening) across tasks in the combined groups

	Cards threatening appraisals	Telepath threatening appraisals	VASP threatening appraisals
Cards threatening appraisals	–	0.70***	0.59***
Telepath threatening appraisals		–	0.61***
VASP threatening appraisals			–
	Cards non-threatening appraisals	Telepath non-threatening appraisals	VASP non-threatening appraisals
Cards non-threatening appraisals	–	0.39**	0.40**
Telepath non-threatening appraisals		–	0.34**
VASP non-threatening appraisals			–

VASP, Virtual acoustic space paradigm.

** *p* < 0.01, *** *p* < 0.001.

### Cards task

In all, one symptomatic (5.55%), three remitted (18.75%), four non-clinical (25%) and 10 control participants (47.62%) guessed the nature of the Cards task correctly. Group was a significant predictor of correct guesses (*χ*^2^ = 9.60, *p* < 0.05), with standardized residuals indicating that controls were more likely than symptomatic and remitted participants to guess the task.

There was a main effect of group for threatening appraisals (Table 5). As predicted, symptomatic participants had higher scores than non-clinical participants and controls, while the latter two groups did not differ. Contrary to predictions, the remitted group had lower (at near-significant level, with a medium effect size) threatening appraisal scores than the symptomatic group, and did not differ from the non-clinical or control groups. There were no group differences for non-threatening appraisals.

**Table 5.** Appraisal scores and global ratings of how striking, threatening and distressing the three experimental tasks were in symptomatic, remitted, non-clinical and control groups, and statistical comparisons (with effect sizes)

	C-S (*n* = 18)	C-R (*n* = 16)	NC (*n* = 16)	Controls (*n* = 21)	Significant group effects	C-S *v*. NC	C-R *v*. NC	NC *v*. controls	C-S *v*. C-R	C-S *v*. controls	C-R *v*. controls
Threatening appraisals											
Cards	2.57 (2.14)	0.96 (1.39)	0.45 (0.75)	0.65 (0.88)	*H* = 13.11, *p* = 0.004	*U* = 214, *p* = 0.002 (*d* = 0.49)	*U* = 171.5, *p* = 0.102, (*d* = 0.34)	*U* = 156, *p* = 0.728, (*d* = 0.07)	*U* = 87, *p* = 0.050, (*d* = 0.39)	*U* = 267.5, *p* = 0.004 (*d* = 0.42)	*U* = 201, *p* = 0.323 (*d* = 0.20)
Telepath	2.33 (1.65)	1.33 (1.35)	0.36 (0.56)	0.71 (0.94)	*H* = 18.12, *p* < 0.001	*U* = 234.5, *p* < 0.001, (*d* = 0.63)	*U* = 184, *p* = 0.035, (*d* = 0.44)	*U* = 138.5, *p* = 0.370, (*d* = 0.18)	*U* = 89, *p* = 0.059, (*d* = 0.38)	*U* = 283, *p* = 0.001, (*d* = 0.50)	*U* = 212.5, *p* = 0.175 (*d* = 0.26)
VASP	2.30 (1.68)	1.58 (1.50)	0.49 (0.78)	0.88 (1.27)	*H* = 16.95, *p* = 0.001	*U* = 229.5, *p* < 0.001, (*d* = 0.59)	*U* = 192.5, *p* = 0.014, (*d* = 0.50)	*U* = 148.5, *p* = 0.554, (*d* = 0.12)	*U* = 105 *p* = 0.187, (*d* = 0.27)	*U* = 277, *p* = 0.003, (*d* = 0.47)	*U* = 232.5, *p* = 0.047, (*d* = 0.38)
Non-threatening appraisals											
Cards	3.94 (1.49)	4.25 (1.53)	4.52 (2.33)	4.81 (1.38)	*H* = 3.75, *p* = 0.290	–	–	–	–	–	–
Telepath	4.56 (2.30)	4.13 (1.44)	3.92 (2.09)	4.40 (1.38)	*H* = 1.07, *p* = 0.785	–	–	–	–	–	–
VASP	5.39 (1.53)	4.81 (1.76)	6.04 (1.28)	6.10 (0.84)	*H* = 7.86, *p* = 0.049	*U* = 106.5, *p* = 0.198, (*d* = 0.26)	*U* = 71.5, *p* = 0.032, (*d* = 0.44)	*U* = 140.5, *p* = 0.404, (*d* = 0.16)	*U* = 116.5 *p* = 0.347, (*d* = 0.19)	*U* = 135.5, *p* = 0.133, (*d* = 0.28)	*U* = 84, *p* = 0.018, (*d* = 0.56)
Global striking											
Cards	6.00 (3.33)	4.88 (3.28)	2.63 (2.66)	4.29 (3.33)	*H* = 8.79, *p* = 0.032	*U* = 224, *p* = 0.004, (*d* = 0.55)	*U* = 180, *p* = 0.051, (*d* = 0.41)	*U* = 117.5, *p* = 0.123, (*d* = 0.30)	*U* = 114.5 *p* = 0.313, (*d* = 0.20)	*U* = 242.5, *p* = 0.133, (*d* = 0.28)	*U* = 184.5, *p* = 0.617, (*d* = 0.10)
Telepath	6.11 (2.72)	5.25 (3.09)	4.06 (3.12)	3.81 (3.25)	*H* = 6.43, *p* = 0.093	–	–	–	–	–	–
VASP	5.83 (2.73)	5.63 (2.66)	3.63 (3.20)	4.10 (2.15)	*H* = 7.02, *p* = 0.071	–	–	–	–	–	–
Global threat											
Cards	2.56 (3.24)	1.19 (1.52)	0 (0)	0.19 (0.51)	*H* = 19.27, *p* < 0.001	*U* = 202.5, *p* = 0.005, (*d* = 0.41)	*U* = 234.5, *p* = 0.015, (*d* = 0.40)	*U* = 144, *p* = 0.476, (*d* = 0.21)	*U* = 120 *p* = 0.422, (*d* = 0.17)	*U* = 277, *p* = 0.012, (*d* = 0.47)	*U* = 192, *p* = 0.040, (*d* = 0.50)
Telepath	1.56 (2.92)	1.88 (2.42)	0.06 (0.25)	0.24 (0.77)	*H* = 10.12, *p* = 0.006	*U* = 185, *p* = 0.164, (*d* = 0.28)	*U* = 187, *p* = 0.026, (*d* = 0.46)	*U* = 161.5, *p* = 0.844, (*d* = 0.04)	*U* = 165 *p* = 0.484, (*d* = 0.15)	*U* = 236, *p* = 0.192, (*d* = 0.25)	*U* = 238.5, *p* = 0.029, (*d* = 0.42)
VASP	2.28 (3.16)	3.50 (3.12)	0.81 (1.60)	1.24 (1.79)	*H* = 7.90, *p* = 0.048	*U* = 179, *p* = 0.237, (*d* = 0.24)	*U* = 191, *p* = 0.017, (*d* = 0.49)	*U* = 141.5, *p* = 0.421, (*d* = 0.16)	*U* = 178.5 *p* = 0.237, (*d* = 0.24)	*U* = 211.5, *p* = 0.530, (*d* = 0.12)	*U* = 236, *p* = 0.037, (*d* = 0.40)
Global distress											
Cards	2.89 (3.58)	1.56 (2.13)	0 (0)	0.67 (1.24)	*H* = 13.43, *p* = 0.004	*U* = 216, *p* = 0.012, (*d* = 0.50)	*U* = 192, *p* = 0.015, (*d* = 0.50)	*U* = 120, *p* = 0.147, (*d* = 0.29)	*U* = 125 *p* = 0.528, (*d* = 0.13)	*U* = 248, *p* = 0.100, (*d* = 0.31)	*U* = 208.5, *p* = 0.217, (*d* = 0.24)
Telepath	1.39 (2.03)	1.75 (2.49)	0.31 (1.01)	0.38 (1.32)	*H* = 12.42, *p* = 0.018	*U* = 199, *p* = 0.059, (*d* = 0.38)	*U* = 171.5, *p* = 0.102, (*d* = 0.34)	*U* = 165, *p* = 0.940, (*d* = 0.04)	*U* = 147.5 *p* = 0.905, (*d* = 0.02)	*U* = 259, *p* = 0.049, (*d* = 0.37)	*U* = 222, *p* = 0.101, (*d* = 0.32)
VASP	2.83 (3.37)	3.69 (3.34)	2.83 (3.37)	1.57 (2.20)	*H* = 5.89, *p* = 0.117	–	–	–	–	–	–

Data are given as mean (standard deviation).

C-S, Symptomatic; C-R, remitted; NC, non-clinical; VASP, Virtual acoustic space paradigm.

There were main effects of group for global ratings of striking, distress and threat (Table 5). Symptomatic participants found the task more striking and distressing than non-clinical participants, but not controls, and more threatening than did controls and non-clinical participants. The remitted group found the task more striking (at trend-level significance) and distressing than non-clinical participants, and more threatening than both non-clinical and control groups. Symptomatic and remitted participants did not differ across all global ratings, nor did non-clinical and control participants.

### Telepath task

Only one non-clinical (6.25%) and one control participant (4.76%) guessed the trick behind the Telepath task correctly. Group was not a significant predictor of correct guesses. There was a significant effect of group for threatening appraisals (Table 5). As predicted, symptomatic participants had higher scores than did non-clinical participants and controls, as did the remitted group compared with the non-clinical group, but not controls. Also as expected, the control and non-clinical groups did not differ. Unexpectedly, remitted participants had lower scores (at trend level) than symptomatic participants, with a medium effect size. There were no group differences for non-threatening appraisals.

There was a main effect of group for global ratings of threat and distress (Table 5), but not striking. The symptomatic group found it more distressing than controls (and at trend level) than non-clinical participants. The remitted group found the Telepath more threatening than the non-clinical group and controls. Remitted and symptomatic participants did not differ on global ratings, nor did controls and non-clinical participants.

### VASP

In all, 12 symptomatic (66.7%), 12 remitted (75%), 15 non-clinical (93.75%) and 21 controls (100%) guessed the nature of the VASP correctly. Group was not a significant predictor of correct guesses. There was a main effect of group for threatening appraisals (Table 5). As predicted, both symptomatic and remitted participants had higher scores than non-clinical participants and controls, and did not differ from each other; nor did the non-clinical and control groups. There was a main effect for non-threatening appraisals, with remitted participants having lower scores than non-clinical participants and controls. There were no other group differences for non-threatening appraisals.

There was a main effect for global ratings of threat, but not striking or distress (Table 5). The remitted group found the VASP more threatening than the non-clinical group and controls. Symptomatic and remitted participants did not differ on global ratings, nor did controls and non-clinical participants.

## Discussion

### Summary of findings

As predicted, clinical participants with ongoing symptoms displayed greater threatening appraisals across all three anomalous experience-inducing tasks, compared with non-need-for-care individuals with PEs and controls, who did not differ from each other. Remitted participants endorsed more threatening appraisals of Telepath and VASP tasks than the non-clinical group, and of the VASP than controls. Symptomatic and remitted participants also generally found the tasks a more globally distressing and threatening experience than controls and non-clinical participants. Furthermore, the two clinical groups did not differ in any global ratings; neither did non-clinical and control participants. Overall these results are consistent with previous findings that clinical status can be differentiated by cognitive appraisals of anomalous experiences. They also bring validity to a novel task, the Telepath, an analogue of thought interference similar to the Cards task. The Telepath produced higher effect sizes than the Cards task when comparing symptomatic participants and non-clinical participants, and fewer participants across groups guessed the manipulation.

**Fig. 1.**
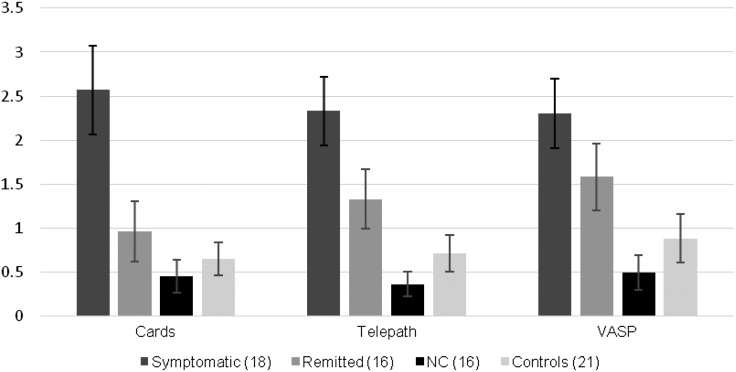
Differences in threatening appraisals scores for each task in symptomatic, remitted, non-clinical (NC) and control groups. Values are means, with standard errors represented by vertical bars. VASP, Virtual acoustic space paradigm.

Contrary to predictions, however, symptomatic and remitted participants differed from one another in terms of threatening appraisals for two out of the three tasks, albeit at trend level and with medium, rather than large, effect sizes. The remitted and control groups also showed statistically indistinguishable threatening appraisals for two of the three tasks. As can be seen clearly in Fig. 1, ratings for the remitted group were intermediate between the symptomatic group and controls for each task, with non-clinical participants having the lowest ratings.

### Implications for the cognitive model of psychosis

In cognitive models of psychosis (Bentall *et al.* 2001, 2007; Garety *et al.* 2001, 2007; Morrison, 2001; Howes & Murray, 2014), threatening appraisals are not considered specific to psychosis, but rather a transdiagnostic risk factor representing cognitive biases and disturbed affect that develop independently of PEs, and are thus trait-dependent (Freeman & Garety, 2003). Although causality cannot be inferred, the findings in the remitted group indicate a potential dampening of threatening appraisals as symptoms remit in clinical individuals, implying that threatening appraisals may be, in part, secondary to the presence of PEs, and thus state-dependent. However, non-clinical participants had significantly more current PEs than did remitted individuals, despite having the lowest threatening appraisal scores of any group. Hence, it is possible that appraisals are both state- and trait-dependent. It may be that clinical individuals exhibit an already heightened threatening appraisal style that becomes exacerbated as symptoms become more frequent and intense. Those without a need-for-care, on the other hand, have managed to decouple threatening appraisals from PEs entirely.

Another explanation may relate to PEs and appraisal styles sharing a dynamic relationship. Studies using experience sampling methods show subtle fluctuations in the relationship between negative affect, stress and symptom intensity (Delespaul & Van Os, 2002; Peters *et al.* 2012; Kramer *et al.* 2014). Successfully decoupling threatening appraisals from PEs may in turn affect their content, frequency and intensity. Potentially, non-need-for-care individuals’ experiences have never reached the same intensity and frequency of those in clinical groups, further facilitating a decoupling of threatening appraisals from their experiences. This could be due to increased striatal dopamine in clinical individuals, producing greater aberrant salience (Kapur, 2003), and thus increased PEs (Howes & Murray, 2014). Interestingly, healthy voice-hearers have been found to show intact dopamine regulation (Howes *et al.* 2013). This suggests, alternatively, that dopamine dysregulation may not drive presence of PEs *per se*, but secondary factors, such as intrusiveness, or even threatening appraisals.

It is also possible that specific types of experiences play a role in eliciting threatening appraisals; for instance, clinical individuals hear voices with greater unpleasant content than healthy voice-hearers (Daalman *et al.* 2011). Given that the VASP produced the largest differences between clinical and non-clinical groups, it may be that auditory hallucinations are a greater predictor of heightened threat appraisal.

There were significant demographic differences between the groups. Estimated IQ in the non-clinical group was higher than in both clinical groups, for example. Such differences have been found consistently across previous studies (Brett *et al.* 2007; Lovatt *et al.* 2010; Ward *et al.* 2014), and may be significant determinants of a need-for-care (Peters *et al.* in press). This would imply that they represent real group differences rather than sampling error, whether these differences are a consequence of group status (e.g. distress, low mood), or developmental risk factors (e.g. low IQ, socio-economic deprivation). Ideally these differences would be controlled for in the analysis. However, there is a compelling argument that statistical methods used to control for group differences, such as analysis of covariance, should only be used to control for random variance, and it is therefore invalid to control for pre-existing, non-random group differences common to psychopathology research (Miller & Chapman, 2001).

Nevertheless, such group differences are likely to be relevant to the ability of non-need-for-care individuals to appraise PEs as non-threatening. There is limited evidence, for example, that exposure to trauma is equivalent between those with and without a need-for-care, but that the specific types and impact of trauma differ significantly (Lovatt *et al.* 2010). Similarly, a recent study showed that the jumping-to-conclusions bias, which potentially underlies threat appraisal, is less pronounced in those without a need-for-care (Lim *et al.* 2012). Future studies should attempt to test whether the relationship between PEs and threatening appraisals is mediated by trauma, IQ, socio-economic status and other environmental factors, to determine which may have a protective effect *v*. those that increase risk for distress.

### Limitations

The remitted and symptomatic groups differed demographically and clinically, with the former group having fewer previous admissions, despite similar onset and duration of illness. The remitted group were also less likely to be diagnosed with schizophrenia, which carries a worse long-term prognosis than affective disorders (Jobe & Harrow, 2005). The differences in severity of illness between the two patient groups may have contributed to the unexpected differences in threatening appraisals.

The symptomatic group had more severe positive symptoms generally than the non-clinical group on the SAPS, suggesting that these groups may not be wholly comparable. However there were fewer differences on the AANEX, a measure more suited for interviewing non-clinical populations than the SAPS. Moreover, the minimum inclusion criteria for the non-clinical group required that PEs occur at least weekly.

Both clinical groups exhibited heightened depression, anxiety and stress scores compared with the non-clinical and control groups, as well as lower IQ scores. Affective symptoms may result in PEs being perceived as more subjectively intrusive, and personally significant (Krabbendam & Os, 2005), and a personalizing bias for negative events has been linked to poor IQ (Berry *et al.* 2014).

Another limitation was the high percentage of participants who guessed the manipulation behind the tasks correctly, particularly in the VASP task. This may have been affected by the number of repetitions, as each task had been altered to be presented multiple times (see Appendix 2). Despite this, there were no significant differences between groups for percentage of correct guesses, apart from the Cards task, where controls were significantly more likely to guess the manipulation. Crucially, each task produced a similar pattern of results, regardless of the percentage of correct guesses, suggesting that correct guesses did not invalidate the findings.

The number of group comparisons being conducted across the three tasks may have inflated type I error. Multiple Mann–Whitney *U* tests were employed as there are no satisfactory non-parametric equivalents of the *post-hoc* Tukey test for individual comparisons. Bonferroni or Dunn–Sidak adjustment is considered an overcautious method that can miss meaningful group differences (Ruxton & Beauchamp, 2008). Coupled with the small sample sizes, it was felt that the risk of type II error outweighed that of type I. Furthermore, effect sizes provide a better indication of real group differences (Kirk, 2003; Cumming, 2013), which in this study were moderate to large overall for the significant and near-significant findings, and negligible for the non-clinical and control group comparisons.

A further limitation is that some of the remitted (*n* = 10; 63%) and symptomatic (*n* = 8; 44%) individuals had received cognitive–behavioural therapy for psychosis (CBTp), which targets threatening appraisals (Fowler *et al.* 1995). Those who had received CBTp in the remitted group (but not the symptomatic group) had lower threatening appraisal scores than those who had not (*U* = 11.5, *p* = 0.042, *d* = 0.62), although only on the Telepath. The high number in the remitted group with prior exposure to CBTp may have contributed to this group's intermediate threatening appraisal scores relative to other groups. The small sample sizes make it difficult to make a proper inference, but the effects of CBTp on appraisals in the clinical groups cannot be discounted. Finally, sampling bias in the way each group was recruited cannot be excluded as a source of variance.

## Conclusions

Overall, this study has provided further evidence that how PEs are interpreted, rather than their presence, may be key to clinical status. Non-clinical participants with PEs were characterized by the lack of threatening appraisals of anomalous perceptual experiences on all tasks, similarly to controls, and unlike psychosis patients.

The novel and unexpected finding was that clinical participants whose symptoms had remitted, a heretofore untested population with regards to appraisals of PEs, exhibited threatening appraisals of the tasks that placed them in between their symptomatic counterparts and controls, implying that threatening appraisals and distressing symptoms may partly diminish in tandem. Nevertheless, those without a need-for-care appraised anomalous experiences as non-threatening despite ongoing PEs, suggesting that they had found a way to decouple threatening appraisals from the presence of PEs. Further research should seek to uncover the potential mediating factors in this relationship.

## Figures and Tables

**Fig. 2.**
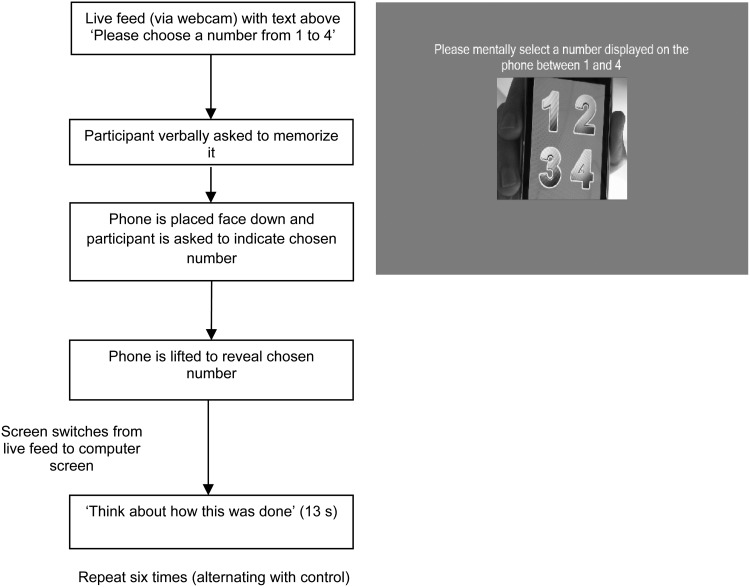
Telepath experimental condition

## Note

1 All three anomalous experience-inducing tasks described were adapted to facilitate future use in a neuroimaging environment, which included repeated exposures and the inclusion of a control condition. The tasks in their adapted forms were piloted and found to have retained their effectiveness. See Appendix 2 for details. Appraisal score data presented herein were in response to these adapted tasks, but for the sake of succinctness the control conditions, which are not relevant to this study, have been omitted from task descriptions.

## Footnotes

† The note appears after the main text.

## Appendix 1 Telepath task description

See Fig. 2 for an illustration of the Telepath task. While the phone was face down, the experimenter waited the requisite amount of time for the desired number to appear on the phone's screen (8 s for ‘1’, 16 s for ‘2’, 24 s for ‘3’, and 32 s for ‘4’). During this time, the experimenter distracted the participant by asking them to focus mentally on their number, rehearse it, and then attempt to transmit it to the device onscreen. As a consequence of the differing lengths of time for each number, the experimental condition did not have a set length.

## Appendix 2 Adaptation of the three anomalous experience-inducing tasks

All three tasks were adapted, so that their adapted forms would lend themselves to usage within a neuroimaging environment. Adapting the tasks involved taking into account the design and presentational constraints imposed by the magnetic resonance imaging scanner, such as stimuli having to be presented on a computer screen, participant responses being non-verbal, and each experimental condition matching with a control condition (not reported in this study) identical in all respects apart from the variable of interest.

Central among these constraints was the number of trials per task, as neuroimaging typically demands multiple presentations of a stimulus in order to yield reliable activation in the region(s) of interest. The most suitable solution (minimum of five trials) was found in a previous study investigating auditory verbal hallucinations in psychotic patients.

Piloting was conducted with five controls and five non-clinical individuals. Overall, the control conditions and multiple trials for each task were not perceived as confusing, and did not contribute to awareness of the manipulation. Additionally, verbal feedback from participants also indicated that the voice samples heard during the VASP were perceived as externally located, even if they knew that it was a recording, thus validating its design.
